# The biomechanical properties of the cornea and anterior segment parameters

**DOI:** 10.1186/1471-2415-13-49

**Published:** 2013-10-02

**Authors:** Ho Sik Hwang, Seh Kwang Park, Man Soo Kim

**Affiliations:** 1 Department of Ophthalmology, Chuncheon Sacred Heart Hospital, College of Medicine, Hallym University, Chuncheon, Korea; 2 BGN Eye Clinic, Seoul, Korea; 3 Department of Ophthalmology and Visual Science, Seoul St. Mary’s Hospital, College of Medicine, Catholic University of Korea, #505 Banpo-dong, Seoul, Korea

## Abstract

**Background:**

To investigate the biomechanical properties of the cornea measured with the Ocular Response Analyzer (ORA) and their association with the anterior segment parameters representing the geometric dimensions including the corneal volume and anterior chamber volume.

**Methods:**

A retrospective review of 1020 patients who visited the BGN Eye Clinic was done. The mean radius of the corneal curvature, corneal astigmatism, corneal volume, anterior chamber depth, and anterior chamber volume were measured with an anterior segment tomographer. The central corneal thickness (CCT) was measured with an ultrasonic pachymeter. The corneal diameter was measured with an Orbscan as White to White. Cornea hysteresis (CH), corneal resistance factor (CRF), Goldmann correlated intraocular pressure (IOPg), and cornea-compensated IOP (IOPcc) were measured with an ORA. Multiple linear regression models were constructed with CH and CRF as the dependent variables and age, gender, and the anterior segment parameters as the covariates.

**Results:**

958 eyes from 958 patients (mean age 26.7 years; male 43.4%) were included in this study after excluding some eyes according to the exclusion criteria. The mean CH and CRF were 10.1 and 9.9 mmHg, respectively. The mean IOPg and IOPcc were 14.8 and 15.8 mmHg. The multivariate analysis showed that CH was negatively associated with the mean radius of the cornea curvature (regression coefficient = - 0.481, p = 0.023) and positively associated with CCT (regression coefficient =0.015, p < 0.001) and corneal volume (regression coefficient =0.059, p = 0.014). The association between CH and the corneal diameter, anterior chamber depth, and anterior chamber volume were not statistically significant. The evaluation of CRF showed that CRF was negatively associated with the mean radius of the cornea curvature (regression coefficient = - 0.540, p = 0.013), and positively associated with CCT (β = 0.026, p < 0.001). The association between CRF and the corneal diameter, corneal volume, anterior chamber depth, and anterior chamber volume were not statistically significant.

**Conclusion:**

The CH was shown to be positively associated with the corneal volume and the association between CH and the anterior chamber volume were not significant. The associations of CRF with the corneal volume or anterior chamber volume were not significant.

## Background

Recently, the Ocular Response Analyzer (ORA; Reichert Ophthalmic Instruments, Buffalo, New York, USA) has enabled ophthalmologists to quantitatively assess the biomechanical properties of the cornea such as the corneal hysteresis (CH) and corneal resistance factor (CRF). The ORA records corneal inward and outward applanation after delivering a metered collimated air pulse and provides an indication of the viscosity and elastic properties of the cornea. CH is thought to predominantly reflect the viscous properties of the cornea, and CRF is an empirically derived measure representative of the cornea’s elastic properties [1]. Unlike the Goldmann tonometer, the ORA produces a cornea compensated intraocular pressure (IOPcc) less influenced by corneal tissue properties. Moreover, corneal biomechanical properties may have great value in the preoperative screening of refractive surgery candidates, helping to differentiate between healthy and abnormal corneas (e.g., Keratoconus and Fuch’s dystrophy) [2-4].

Up to now, there have been some studies on the biomechanical properties of the cornea measured by the ORA and their association with anterior segment parameters including the mean radius of the corneal curvature, corneal astigmatism, central corneal thickness, corneal diameter, and anterior chamber depth [5-14]. The central corneal thickness was positively correlated with CH and CRF in many studies, but the other parameters have controversial correlation with CH and CRF.

An anterior segment tomographer (e.g., Pentacam) can measure the cornea volume and anterior chamber volume with Scheimpflug images besides the aforementioned anterior segment parameters [15]. Both CH representing corneal viscosity (energy absorption) and CRF representing elasticity of the cornea may have significant associations with the corneal volume and anterior chamber volume. However, to the best of our knowledge, there is no other study on the association between CH and CRF and the corneal volume and anterior chamber volume, respectively. Therefore, the aim of this study was to investigate the biomechanical properties of the cornea measured with the ORA and their association with the anterior segment parameters representing geometric dimensions such as corneal volume and anterior chamber volume.

## Methods

### Subjects

A retrospective review of 1020 patients who visited the BGN Eye Clinic (Seoul, Korea) for refractive surgery from January to December in 2011 was done. The study followed the principles of the Declaration of Helsinki and the Institutional Review Board of Seoul St. Mary’s Hospital (Seoul, Korea) approved the study.

### Measurements

Each subject underwent a comprehensive ophthalmologic examination, which included a medical history review, best-corrected visual acuity (BCVA), and slit-lamp and fundoscopic examinations. Refractive error was measured by Autoref-Keratometer (Canon RK-5, Autoref-Keratometer, Canon Inc. Ltd., Japan). The mean radius of the corneal curvature, corneal astigmatism, corneal volume, anterior chamber depth, and anterior chamber volume were measured with a Pentacam (Oculus Pentacam Rotating Scheimpflug Camera; Oculus, Wetzlar, Germany). The central corneal thickness was measured with an ultrasonic pachymeter (Model US-1800; Nidek Co LTD, Gamagori, Japan). The corneal diameter was measured with an Orbscan (Orbscan IIz, Bausch & Lomb GmbH, Feldkirchen, Germany) as White to White.

CH, CRF, Goldmann correlated IOP (IOPg), and IOPcc were measured with an ORA. The ORA generates 2 IOP measurements (*P*1 and *P*2). The difference between *P*1 and *P*2 is the measure of the CH. The IOPg is only the average of P1 and P2. The IOPcc is generated with software and it represents an IOP less influenced by the corneal tissue properties. The CRF is derived from software with the following formula: CRF = *P*1 - *kP*2, where *k* is a constant that has been determined from an empirical analysis of the relationship between *P*1 and *P*2 and CCT [1]. Details regarding the ORA have been published previously[9]. The ORA parameters were measured in the right eye of each eligible subject. Patients were seated on a chair and instructed to place their foreheads on the headrest of the ORA device. The ORA parameters were collected consecutively and only good readings were used, as defined by both the in and out applanation signal peaks on the waveforms being symmetrical. The mean value of three measurements was taken. The exclusion criteria for this analysis were a history of intraocular surgery, refractive surgery, contact lens use within 2 weeks, the presence of corneal abnormalities such as keratoconus, corneal scarring that would preclude accurate ORA and IOP measurements, or a diagnosis of “glaucoma suspect” or glaucoma. Eyes in which the IOPg or IOPcc were more than 21 mmHg were also excluded in this study.

### Statistical analysis

The mean values of CH and CRF and their associations with the anterior segment parameters were analyzed. A histogram showing the distribution of CH and CRF was generated. Multiple linear regression models were constructed with CH and CRF as the dependent variables and age, gender, and the anterior segment parameters as the covariates. Analyses were done with the statistical software, SPSS version 15 (SPSS Inc. Chicago, IL, USA). In consideration of multiple testing problems, a *p* value < 0.025 was considered statistically significant for two primary outcomes, CH and CRF.

## Results

A total of 1020 eyes from 1020 Koreans were originally included in the study. Eventually, 958 eyes from 958 patients were included in this study after excluding some eyes according to the exclusion criteria. Sample size of 958 eyes has power of the test larger than 85% in multivariate analysis with seven independent variables, small effect size, f^2^ = 0.02 (R^2^ = 1.96%) and level of significance 0.025 [16].

**Figure 1:**
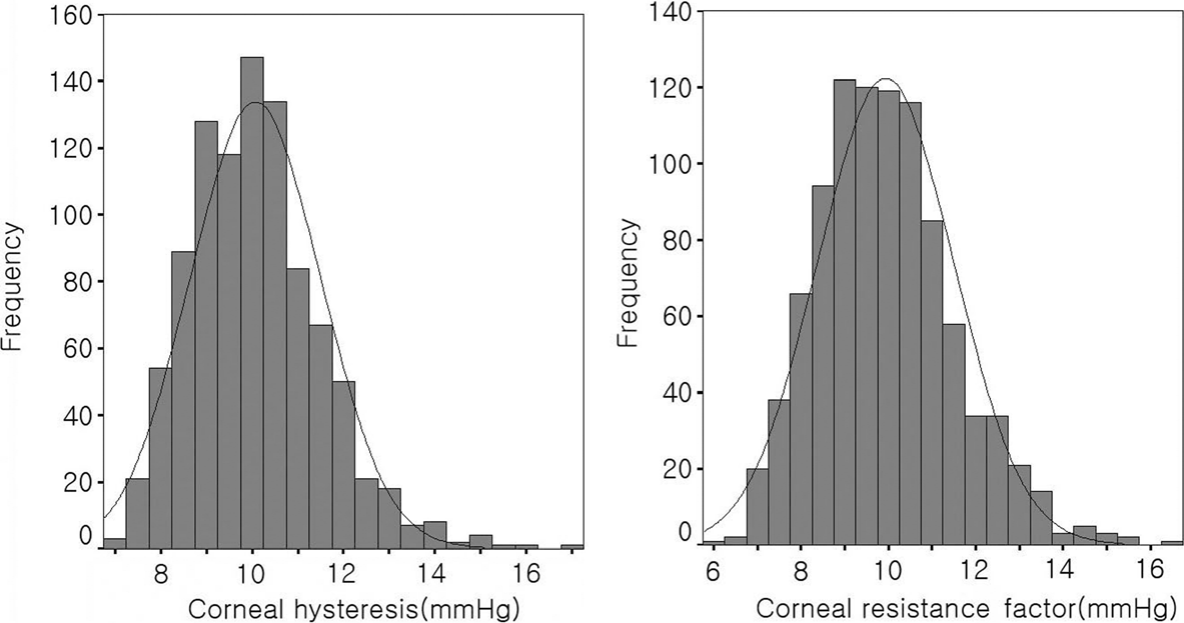
Distribution of corneal hysteresis and corneal resistance factor in Korean.

Table 1 summarizes the mean values of the ocular variables of the study population. The mean age of the patients was 26.7 ± 6.1 years. Four hundred and sixteen (43.4%) of them were male and 542 (56.6%) of them were female. The mean corneal volume was 63.0 ± 3.3 mm^3^ and the mean anterior chamber volume was 192 ± 31 mm^3^. The mean CH was 10.1 ± 1.4 mmHg, and the mean corneal resistance was 9.9 ± 1.6 mmHg. Figure 1 is a histogram of the distribution of CH and CRF.

**Table 1:** Characteristics of the study population (n = 958)

**Characteristics**	**Mean ± SD or number (%)**	**Range**
Age, y	26.7 ± 6.1	18–54
Gender, male	416 (43.4)	-
Female	542 (56.6)	-
Spherical equivalent, D	-4.7 ± 2.2	-12.1 to +6.5
Mean radius of corneal curvature, mm	7.76 ± 0.25	7.02–9.68
Corneal astigmatism, D	1.35 ± 0.72	0.00–4.40
Central corneal thickness, μm	536 ± 30	428–640
Corneal diameter, mm	11.5 ± 0.34	10.5–13.4
Corneal volume, mm^3^	63.0 ± 3.3	53.7– 73.4
Anterior chamber depth, mm	3.23 ± 0.27	2.24–5.24
Anterior chamber volume, mm^3^	192 ± 31	103–295
Corneal hysteresis, mmHg	10.1 ± 1.4	7.1–17.2
Corneal resistant factor, mmHg	9.9 ± 1.6	6.2–16.4
IOPg^*^, mmHg	14.8 ± 2.6	6.4–20.9
IOPcc^†^, mmHg	15.8 ± 2.5	4.3–20.9

^*^Goldmann correlated intraocular pressure.

^†^Cornea compensated intraocular pressure.

**Figure 2:**
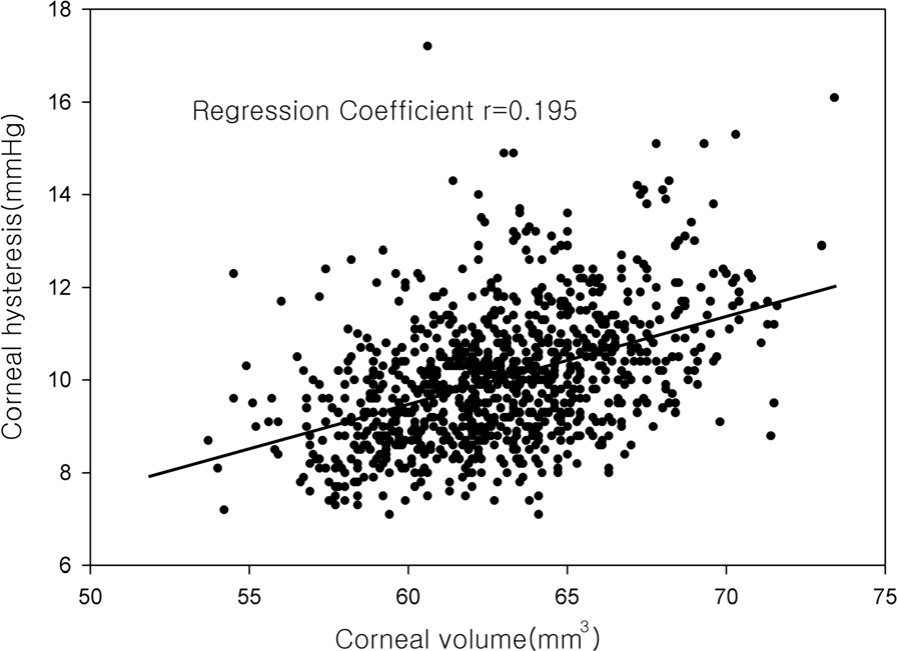
Association between corneal hysteresis and corneal volume.

The associations between the corneal biomechanical parameters with the anterior segment parameters through age- and gender-adjusted linear regression analyses and multivariate models were investigated. After controlling for age and gender, both CH and CRF had statistically significant correlation with the mean radius of corneal curvature, central corneal thickness, corneal diameter, corneal volume, anterior chamber depth and anterior chamber volume (Tables 2 and 3). These parameters were included as covariates in multiple linear regression analyses. The evaluation of CH showed that CH was negatively associated with the mean radius of the cornea curvature (regression coefficient = -0.481, *p* = 0.023) and positively associated with the corneal thickness (regression coefficient = 0.015, *p* < 0.001) and corneal volume (regression coefficient = 0.195, *p* = 0.014) (Figure 2). CRF was negatively associated with the mean radius of the cornea curvature (regression coefficient = -0.540, p = 0.013), and positively associated with the corneal thickness (regression coefficient = 0.026, p < 0.001).

**Table 2:** Linear regression analyses on corneal hysteresis

	**Corneal hysteresis, mmHg**					
	Age-gender model^*^			**Multivariate model**^**†**^		
	**B**	**Standard error**	***p*-value**	**B**	**Standard error**	***p*-value**
Age, y	0.014	0.008	0.070	-		
Gender, male	-0.154	0.093	0.099	-		
Mean radius of corneal curvature, mm	-0.672	0.189	<0.001	-0.481	0.211	0.023
Corneal astigmatism, D	0.108	0.065	0.096	-		
Central corneal thickness, μm	0.021	0.001	<0.001	0.015	0.002	<0.001
Corneal diameter, mm	-0.760	0.135	<0.001	-0.126	0.149	0.398
Corneal volume, mm^3^	0.195	0.013	<0.001	0.059	0.024	0.014
Anterior chamber depth, mm	-0.703	0.180	<0.001	-0.164	0.290	0.573
Anterior chamber volume, mm^3^	-0.009	0.002	<0.001	-0.004	0.003	0.180

^*^Adjusted for age and gender.

^†^Adjusted for mean radius of corneal curvature, central corneal thickness, corneal diameter, corneal volume, anterior chamber depth and anterior chamber volume; adjusted r^2^ = 0.230.

**Table 3:** Linear regression analyses on corneal resistance factor

	**Corneal resistant factor, mmHg**					
	Age-gender model^*^			**Multivariate model**^**†**^		
	**B**	**Standard error**	***p*-value**	**B**	**Standard error**	***p*-value**
Age, y	0.006	0.008	0.488	-		
Gender, male	-0.075	0.102	0.462	-		
Mean radius of corneal curvature ,mm	-0.503	0.208	0.016	-0.540	0.216	0.013
Corneal astigmatism, D	0.098	0.071	0.166	-		
Central corneal thickness, μm	0.029	0.001	<0.001	0.026	0.003	<0.001
Corneal diameter, mm	-0.759	0.149	<0.001	-0.101	0.153	0.510
Corneal volume, mm^3^	0.239	0.013	<0.001	0.029	0.025	0.244
Anterior chamber depth, mm	-0.767	0.197	<0.001	-0.234	0.298	0.433
Anterior chamber volume, mm^3^	-0.010	0.002	<0.001	-0.002	0.003	0.465

^*^Adjusted for age and gender.

^†^Adjusted for mean radius of corneal curvature, central corneal thickness, corneal diameter, corneal volume, anterior chamber depth and anterior chamber volume; adjusted r^2^ = 0.324.

## Discussions

This study investigated the biomechanical properties of the cornea measured with an ORA and their association with the anterior segment parameters including the mean radius of the corneal curvature, corneal astigmatism, central corneal thickness, corneal diameter, corneal volume, anterior chamber depth, and anterior chamber volume of 958 eyes from 958 Koreans. In this study, we found, for the first time, from the multivariate analysis that CH was positively associated with the corneal volume and the association between CH and the anterior chamber volume was not significant. The associations between CRF and the corneal volume or the anterior chamber volume were not significant. Moreover, the finding that that cornea diameter and corneal astigmatism have no significant association with CH and CRF was different from those of a previous study [10].

CH is a measure of the energy absorption during the loading/unloading of the stress–strain cycle of viscoelastic materials [8]. CRF, on the other hand, is heavily weighted by elasticity because it was designed for maximum correlation with corneal thickness [17].

In this study, the mean radius of the corneal curvature was negatively correlated with CH and CRF. In other words, the steeper the cornea was, the larger the CH and CRF were. Results in studies in which dynamic contour tonometry was used have suggested that corneal curvature affects corneal rigidity, with steeper corneas being more rigid [18,19]. The more rigid corneas showed higher CH and CRF values. This finding, especially, is in agreement with the fact that the CRF, which represents the corneal elasticity, has a larger value with a steep cornea. In some studies, the mean radius of the corneal curvature was negatively correlated with CH and CRF just as in our study [9,11], and in some studies, there was no significant association between them [5,6]. This difference could be related to the small sample size in the previous studies or could represent ethnic variations in biomechanical properties.

There was only one report on the association of corneal astigmatism with CH and CRF [10]. The report showed that corneal astigmatism was negatively correlated with CH and CRF. However, they included only 50 French subjects in their study. In this large-scale study, there was no significant association between them. This difference could be related to the small sample size or could represent ethnic variations in biomechanical properties.

The central corneal thickness was positively correlated with CH and CRF in many studies [5-9,11-14], and this is consistent with the findings in this study. It is obvious that CRF was positively correlated with the central corneal thickness, because CRF was designed for maximum correlation with corneal thickness [17]. It may be due to the fact that the viscosity and elasticity increase as the corneal thickness increases.

There was only one report on the association of the corneal diameter with CH and CRF [10]. They showed that corneal diameter was negatively correlated with CH and CRF. However, in this large-scale study, there was no significant association between them. This difference could be related to the small sample size.

Until now, there have been no studies on the association of the corneal volume with CH and CRF. The corneal volume was positively correlated with CH, but there was no significant correlation between CRF and the corneal volume. The corneal volume may be associated with the energy absorption capacity. Therefore, it is in agreement with the fact that the CH representing the energy absorption and the corneal volume have a positive correlation.

The anterior chamber depth had no significant correlation with CH and CRF. In some studies, the anterior chamber depth had a negative correlation with CH but no significant correlation with CRF [5,11]. Until now, there have been no studies on the correlation of the anterior chamber volume with CH and CRF. The anterior chamber volume had no significant correlation with CH or CRF in this study. Since water volume in the anterior chamber will rarely change with a change in pressure, the anterior chamber depth or volumes may have no correlation with the viscosity and elasticity of the anterior segment of the eye including the cornea.

In Multivariate analysis, both CH and CRF had larger values with steep and thick corneas. It was well known that the IOP measured by the Goldmann tonometer was overestimated with a steep cornea [20]. In addition, variations of CCT in normal corneas can lead to false high-pressure readings with thicker corneas and false low-pressure readings with thinner corneas [21]. Therefore, a future study should investigate the associations between and CH, CRF, IOPg and actual IOP.

In multiple linear regressions of CH and CRF, the coefficient of determination (the r^2^ value) was 0.23 and 0.32, respectively. In other words, 20-30% of the variance of CH and CRF can be explained by variations in the anterior segment parameters representing the geometric dimensions. The other 70-80% could be explained by the innate corneal properties (e.g., the collagen fiber layer structure in the stroma, the composition of the extracellular matrix, and water content). There are many studies that show keratoconus had a lower CH than normal corneas [4,17].

A limitation of the study is the inclusion of young only patients who are not representative of the normal Korean population and may have caused selection bias. However, in another large-scale study, of the determinants of corneal biomechanical properties in an adult Chinese population, CH was negatively associated with corneal radius of curvature and positively associated with CCT [11]. The CRF showed a negative association with corneal radius of curvature, and was positively associated with CCT. These are consistent with our results.

## Conclusions

In summary, in this study, which included 958 eyes from 958 Koreans, through multivariate analysis, we showed the associations between the biomechanical properties and the anterior segment parameters representing the geometric dimensions. CH was shown to be positively associated with the central corneal thickness and corneal volume and negatively associated with mean radius of corneal curvature. CRF was shown to be positively associated with the central corneal thickness and negatively associated with mean radius of corneal curvature.

## Competing interests

The authors declare that they have no competing interests.

## Authors’ contributions

Conceived and designed the study: HSH SKP MSK. Gathered the data: HSH SKP. Analyzed the data: HSH SKP MSK. Wrote the paper: HSH MSK. All authors read and approved the final manuscript.

## Pre-publication history

The pre-publication history for this paper can be accessed here:

http://www.biomedcentral.com/1471-2415/13/49/prepub
